# Application of Wigner Distribution Function for THz Propagation Analysis

**DOI:** 10.3390/s22010240

**Published:** 2021-12-29

**Authors:** Michael Gerasimov, Egor Dyunin, Jacob Gerasimov, Johnathan Ciplis, Aharon Friedman

**Affiliations:** Department of Electrical and Electronic Engineering, Ariel University, Ariel 40700, Israel; edyunin@gmail.com (E.D.); jacobgr@ariel.ac.il (J.G.); yehonatanci@ariel.ac.il (J.C.); aharonfr@ariel.ac.il.com (A.F.)

**Keywords:** Wigner distribution function, THz analysis, transmission line

## Abstract

The construction of a transmission line (TL) for a wide tunable broad-spectrum THz radiation source is not a simple task. We present here a platform for the future use of designs of the TL through our homemade simulations. The TL is designed to be a component of the construction of an innovative accelerator at the Schlesinger Family Center for Compact Accelerators, Radiation Sources and Applications (FEL). We developed a three-dimensional space-frequency tool for the analysis of a radiation pulse. The total electromagnetic (EM) field on the edge of the source is represented in the frequency domain in terms of cavity eigenmodes. However, any pulse can be used regardless of its mathematical function, which is the key point of this work. The only requirement is the existence of the original pulse. This EM field is converted to geometric-optical ray representation through the Wigner transform at any desired resolution. Wigner’s representation allows us to describe the dynamics of field evolution in future propagation, which allows us to determine an initial design of the TL. Representation of the EM field by rays gives access to the ray tracing method and future processing, operating in the linear and non-linear regimes. This allows for fast work with graphics cards and parallel processing, providing great flexibility and serving as future preparation that enables us to apply advanced libraries such as machine learning. The platform is used to study the phase-amplitude and spectral characteristics of multimode radiation generation in a free-electron laser (FEL) operating in various operational parameters.

## 1. Introduction

In recent years, significant progress was made in the development of terahertz radiation sources. Studies have shown that THz radiation is environmentally friendly and since it is not ionizing, and not dangerous to biological subjects [1,2].

Terahertz (THz) radiation refers to electromagnetic waves and in the (0.3–3.75) THz region. The corresponding wavelengths are (1000–80) µm. Terahertz waves can be transmitted through various types of media including paper, plastics, ceramics, wood, and textiles. They enable non-destructive detection of hidden internal substances and are expected to lead to the development of novel methods of non-destructive analysis. Thus, THz research and applications have become widely popular and accessible [3,4]. At the same time, the propagation of THz radiation is not straightforward. It has the properties of an electromagnetic wave on the one hand and the properties of light waves on the other. One recent progress of the THz application in the classic method for computing EM wave problems is the transfer matrix method [5].

Calculating the transmission of a THz signal from a source is not trivial. The main motivation of this work is a characterization of early transmission line (TL) planning of the super-radiant THz free-electron laser (FEL) in the Schlesinger Center, Ariel University [6]. The FEL is under development. The design of the TL will depend on the results of the simulations. The general approach so far has been physically physical. That is to say, a physical problem was defined and there was an attempt to solve it by analytical means. There was then an attempt to use commercial software like CST, which shall be detailed below, but no desired result could be achieved. Furthermore, it was difficult to even define a specific problem. This is because these contents give a limited solution to general problems, which are usually defined mathematically, but their solution is complicated or a numerical solution is needed. The pulses in the scale of terahertz, which the accelerator is supposed to produce, have no defined mathematical function, even approximately. They do not have the desired distribution, like Gaussian for example. Despite its main spine, there is a need to move the whole ‘unclean’ pulse with as minimal an energy loss as possible along with the TL. Before building the TL and ordering the existing T-Hertz mirrors on the market, it was decided to do so in a simulation, so as not to waste resources. After experiments with an undulator (wiggler), a model of approximation of the pulse to a mathematically defined function was proposed (detailed in Section 3). Based on these results, an approximate function was obtained. The research from here was supposed to continue with simulations, using Wigner transformation via an analytical or numerical solution, with the point is that there is a mathematical definition of an input function. Indeed, every pulse in a reality is completely different from its predecessor and there is no approximate distribution. Therefore, the purpose of this work is to develop a tool for calculating the Wigner function [7] for each pulse, regardless of whether it is defined mathematically or not, what distribution it has, etc. The main point is to have an electromagnetic (EM) pulse. This is a significant advancement in research that considers real pulses, with an emphasis on the characterization of the EM field.

The ultimate goal, in particular, for which the code is developed here is to create mode conversion mirrors using artificial intelligence (AI) methods. Nowadays, they are also gaining momentum for predicting wireless signal propagation using ray-tracing (RT) algorithms [8]. This allows for the acceptance of spatial mapping and visualization of the EM field in 5G technology [9]. Using the CNN method with wavelet transform into a spectral representation of cosmic rays (composed of high-energy particles) obtained a very good recognition ratio by amplifying distinctive image features [10].

AI methods are better known as techniques of machine learning (ML) and have been widely used in THz imaging and time-domain spectroscopy [11]. In this work, the emphasis is on spatial-frequency representation of the EM field: when it will be possible to convert the field shape to the desired shape with a minimum of energy loss along the optic way. This means that we change the functions of the mirrors so that as many rays as possible are transmitted in the middle to the target. It also means that we will cause the field to be propagated in a determined way. Therefore, a standard method, which is commonly used, will not work because no approach can satisfy this need. However, for the sake of comparison and alignment, we will note the task (expression) in general through the classical methods.

**Figure 1:**
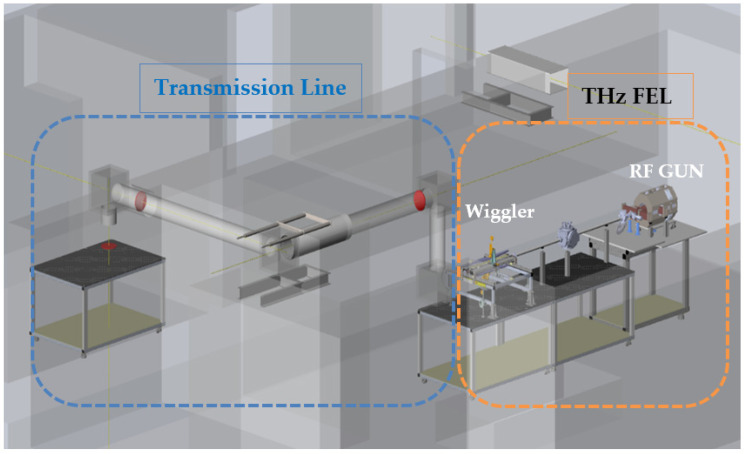
THz FEL setup at the Schlesinger Center.

The FEL is driven by short electron pulses, produced by a photo-injector rf-LINAC gun. Note that a hybrid rf-LINAC structure has both standing and traveling wave sections, which enable the production of short (picosecond or sub-picosecond scale long) electron beam pulses with a controlled energy chirp. It will emit a THz-radiation from a 7.5 × 5 mm flat aperture. The general layout of the THz FEL is shown in Figure 1. The THz radiation spectra may be tuned in the range 1–3.5 THz and have a frequency spread of 10% around the central frequency. The radiation power is distributed in general in three waveguide modes [12].

### 1.1. Review of Simulation Methods

Electrodynamic and optical methods are the most popular technologies for the solution of radiation propagation problems. Analytical electrodynamic methods solve a limited number of idealized geometrical configurations. Numerical electrodynamic methods are based on a finite-difference time-domain approach to Maxwell’s equations, in integral or differential form. Computational electromagnetics (CEM) is applied to model the interaction of electromagnetic fields using Maxwell equations with the deferent objects like an antenna, waveguide, aircraft, and their environment [13].

Optical methods are utilized to find a solution for the wave equation. These are based on ray-representation [14] or Green-function integration [15]. In some cases, di (such as a Gaussian beam) may be applied. The modal approach is aimed at modeling optical-wave propagation in long structures such as fibers or directional couplers, wherein the energy flows primarily along a single principal direction (paraxial approximation) [16]. Gaussian optic does not work well since the diffraction is too large.

A solution for electrodynamic problems requires the definition of the boundary conditions. Electromagnetic field solvers include CST, HFSS, or finite difference time domain (FDTD). A direct solution of Maxwell equations is aimed at modeling compact structures having energy flow in arbitrary directions. FDTD achieves precision by directly solving for fundamental quantities the electric and magnetic (E and H, respectively) fields in space and time rather than performing asymptotic analyses or assuming paraxial propagation. As a result, it is almost completely general. It permits accurate modeling of a broad variety of dispersive and nonlinear media used in emerging technologies such as micron-sized lasers and optical switches [11].

In the range of THz, FDTD comes shortly due to the short wavelength resulting in the grids becoming inhibitive large and requiring extensive computer resources (power and memory, since a large number of mesh elements and samples must be used; see below for example). In addition, the accumulative computation errors may become too large.

### 1.2. Purpose

**Figure 2:**
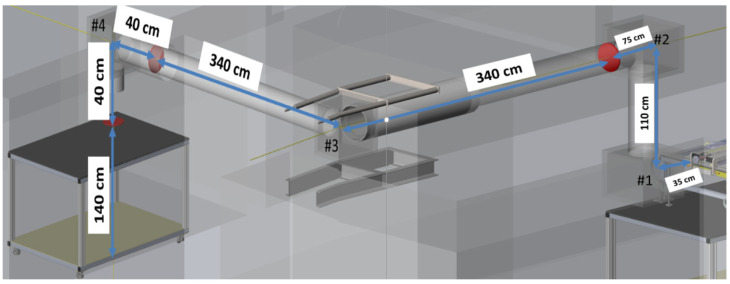
THz transmission line.

The emitted radiation will be transmitted from the source to the lab measurement facility via a 30 cm pipe. The distance between the waveguide’s edges to the user-room is about 10 m as shown in Figure 2. The transmission line is designed to be based on four off-axis parabolic mirrors. All the mirrors are confocal. The first pair will be with focal lengths of F1/F2 = 200/450. The second pair will be with focal lengths of F3/F4 = 1550/350.

In this article, characterization of the field, size (magnitude), and shape was computed. In addition, the spatial and (temporal) distribution of frequencies indicates the concentration or decentralization of energy and allows the examination of the future evolution of the field. The emphasis on field characterization facilitates examining the correctness of TL’s early design. The mirror and TL’s exact design go beyond the scope of this work. We will only mention here the fact that both the transmission line and profile of mirrors at the desired resolution can be performed using this work.

Using electromagnetic solvers and algorithms (such as FDTD) requires at least 10 space samples per wavelength in each dimension. This means that the space mesh-cell size (Δr) is about 10 µm. The time-step size (Δt) is correlated to the space mesh-cell size with the following relationship

$$\Delta r>>c_{0}\Delta t$$

where $c_{0}$ is the speed of light in a vacuum. Thus, the solution of the problem should require about 10^17^ space-mesh points and 10^6^ time steps.

Gaussian optics to THz results can be applied in a Rayleigh length ranging from 10 to 30 cm after the waveguide. However, the distance to the first mirror is about 50 cm due to mechanical limitations. This is compounded by the fact that the field distribution at the waveguide edge cannot be described by a single Gaussian mode.

The propagation of the known field distribution over the aperture in space can be solved by various approaches. For example, following Huygens’s principle [17,18], the field on the aperture may be replaced by the distribution of point sources of spherical waves. The field in the space is defined as a sum of the spherical waves from the distribution of point-like sources. This method may always be applied, but the convergence velocity is very small.

In some cases, the field on the aperture is represented by a sum of Hermite-Gaussian modes [19,20]. Thus, the propagated field in the space is calculated as a sum of the propagated modes [21]. The method demonstrates a good convergence for the case when the initial distribution and the modes have similar behavior on the aperture boundaries (or on the infinity, when the aperture is not limited by a fixed boundary) [22]. However, since the Hermite-Gaussian modes are a paraxial approximation, this method deviates from reality the further we get away from the source.

The mathematical basis for the Huygens principle is the Green function. For us, it would be equivalent to a pack of rays on the initial aperture coming out of each point.

Since the purpose of the article is not to investigate the methods of representation and solution of the electromagnetic field, no additional methods will be specified here. From now on, there will be a focus on the chosen method, which will be explained in the next paragraph.

In light of the fact that the FEL is under development, the transmission line is not built at all, which does not allow us to do a physical experiment. Therefore, the simulations are supposed to provide a solution for the transmission line design.

## 2. Wigner Methodology

The Wigner distribution function (WDF) is a phase-space representation that was used in optics over 30 years ago [23]. Originally, it was introduced by E. Wigner [24] as the simplest quantum analogue of the classical phase space distribution function allowing to find the probabilities and mathematical expectations of quantum operators in as classical a way as possible.

Since then, it has been utilized in numerous applications and has become a very useful tool for the theoretical analysis of optical [25] (including quasi-optical) systems, acoustics [26], signal processing [27], radio waves used in wireless technology [28], etc. Furthermore, it is used to represent the characteristics of radar emitters [29]. The vast majority of the works with the WDF relate to time-frequency analysis [30]. In this work, spatial frequencies are of greater importance.

Here we will advocate a general approach to electromagnetic fields (EM), especially an inexact description of free electromagnetic wave fields in terms of rays [31] and with emphasis on THz radiation systems (see [32] and references therein).

WDF is one of the fairly new ways of characterizing an electromagnetic field in general and a unified description of partially coherent optical beams in particular. The WDF explores the concept of phase space. The evaluation of most important beam parameters (such as intensity, phase coherence, beam-width, etc.) during the propagation, following T. Alieva etc. [33], can be described by the pure space or the pure spatial-frequency representation of a stochastic process via its mutual intensity (MI).

By the MI, such beams are easily described with $\Gamma \left(r_{1},r_{2}\right)=\langle f\left(r_{1}\right)f$*$\left(r_{2}\right)\rangle$ which is a function of four variables: the vectors ***r***_1_ and ***r***_2_. The brackets for time or spatial averaging and the asterisk * means a complex conjugate. In our case, the MI describes the modified correlation between the EM field oscillations at two points ***r***_1_ and ***r***_2_. Note that $\Gamma \left(r,r\right)=\langle |f\left(r\right){|}^{2}\rangle$ corresponds to the intensity distribution ***I***(**r**)**,** and so can be measured.

We can define the WDF of an EM Field W(r,p) by its MI and also in terms of its directional spectrum. It equals [33]

(1) $$W\left(r,p\right)=\int \Gamma \left(r+\frac{\tilde{r}}{2},r-\frac{\tilde{r}}{2}\right)e^{\left(-i2\pi p\tilde{r}\right)}d\tilde{r}$$

(2) $$W\left(r,p\right)=\int \bar{\Gamma }\left(p+\frac{\tilde{p}}{2},p-\frac{\tilde{p}}{2}\right)e^{\left(+i2\pi r\tilde{p}\right)}d\tilde{p}$$

This space-frequency description is reminiscent of the “Ray Concept” in Geometrical Optics (GO), or ray optics that is defined by the position and direction of the ray. ***W***(***r*, *p***) is the amplitude of a ray passing through the point ***r*** with a frequency ***p*** (i.e., direction *q*).

It was shown [32] that the electromagnetic field propagation in the space from a given aperture may be represented by a set of GO rays defined by the Wigner distribution. Let us concentrate on the case of the flat aperture, which is oriented in space perpendicular to the *z*-axis. For a known monochromatic field distribution on the aperture, the Wigner distribution is defined by

(3) $$W_{\left\{x,y\right\}}{\left(x,y,k_{x},k_{y}\right)|}_{z_{aperture}}=\iint E_{\left\{x,y\right\}}^{*}{\left(x-\frac{\tilde{x}}{2},y-\frac{\tilde{y}}{2}\right)|}_{z_{aperture}}E_{\left\{x,y\right\}}{\left(x+\frac{\tilde{x}}{2},y+\frac{\tilde{y}}{2}\right)|}_{z_{aperture}}e^{\left(+ik_{x}\tilde{x}\right)}\cdot e^{\left(-ik_{y}\tilde{y}\right)}d\tilde{x}d\tilde{y}$$

where $W_{\left\{x,y\right\}}$ are the WDF of $E_{\left\{x,y\right\}}$ EM field, *x*, *y* are the coordinates on the aperture surface, and $k_{x},k_{y}$ are Fourier conjugated variables to the space coordinates. Considering the time-dependence of the EM-field transfers, the WDF equations from 4D to 6D-function. In this paper, we concentrate on single-frequency problem.

As the result, the rays from each point *xy* on the aperture are obtained. The direction of each ray is defined by the wave number $k=(k_{x},k_{y},k_{z})$ where

(4) $$k_{z}=\sqrt{{\left(\frac{\omega }{c}\right)}^{2}-k_{x}^{2}-k_{y}^{2}}$$

The ray amplitude and polarization (***I***) is defined by the Wigner distribution

(5) $$I_{x}\left(x,y,k_{x},k_{y}\right)=W_{x}\left(x,y,k_{x},k_{y}\right)$$

(6) $$I_{y}\left(x,y,k_{x},k_{y}\right)=W_{y}\left(x,y,k_{x},k_{y}\right)$$

Due to the orthogonality in any uniform media of the ray polarization to the ray wavenumber (I⊥k)

(7) $$I_{z}=\{\begin{array}{ll} -\frac{I_{x}\cdot k_{x}+I_{y}\cdot k_{y}}{k_{z}} & k_{z}\neq 0\\ 0 & k_{z}=0 \end{array}$$

Thus, in each point (*x,y*) on the aperture with the given field distribution (***E***) we obtain the set of GO rays ***I***$(x,y,k_{x},k_{y}$). The signal propagation in the space after the aperture is represented by the propagation of the GO rays [34].

## 3. Results

### 3.1. Previous Investigations on EM-Field Distribution on the Aperture

#### 3.1.1. Previous Investigations of the THz Radiation

Previous investigations of the THz radiation generated in a super-radiant modern THz free-electron laser (FEL) by an electron beam going through a planar wiggler results in the electric field distribution in the waveguide [35]. The model is based on a coupled-mode approach expressed in the frequency domain [36]. The WB3D particle simulation code is used to calculate the total electromagnetic field excited by an electron beam transported along a waveguide in the presence of a wiggler field of FEL.

The code is based on the space-frequency 2D approach described in more detail in [37]. Expansion and simulation, including the WB3D code results of the electromagnetic pulse generated by FEL in the waveguide, will be explained in the next paragraph.

#### 3.1.2. FEL Simulations

Simulations of the radiation pulse at the output of the Israeli THz free-electron radiation source were developed at Ariel University [6]. They were carried out in the framework of the self-consistent, space-frequency approach as a description of the pulsed relativistic electron beam propagation through a magnetostatic structure and its radiation emission [38]. The approach was realized in the numerical code WB3D, and has been successfully applied for analysis of various processes taking place in free-electron radiation sources (see [12,39], and references therein).

Primarily, the WB3D code was applied to the analysis of the radiation excitation and the beam propagation in the source. The code is supposed to utilize short, energy chirped relativistic electron bunches obtained from a hybrid laser-driven photo-injector. Such a configuration enables an effective production of strong pulses of terahertz radiation emitted thanks to the enhanced super-radiation mechanism [12,40].

**Figure 3:**
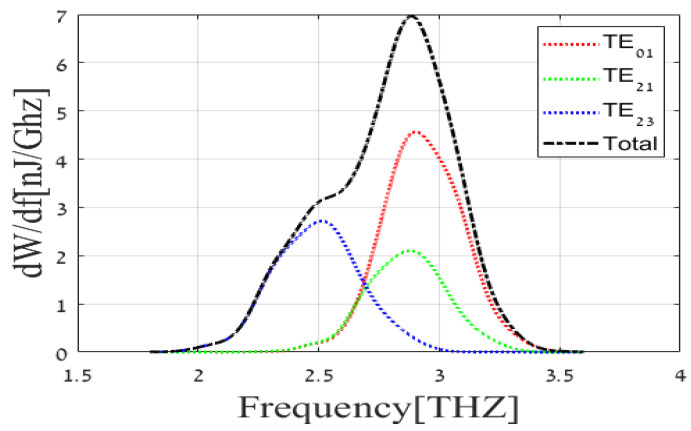
Output amplitude radiation spectrum of Israeli free-electron THz source.

**Figure 4:**
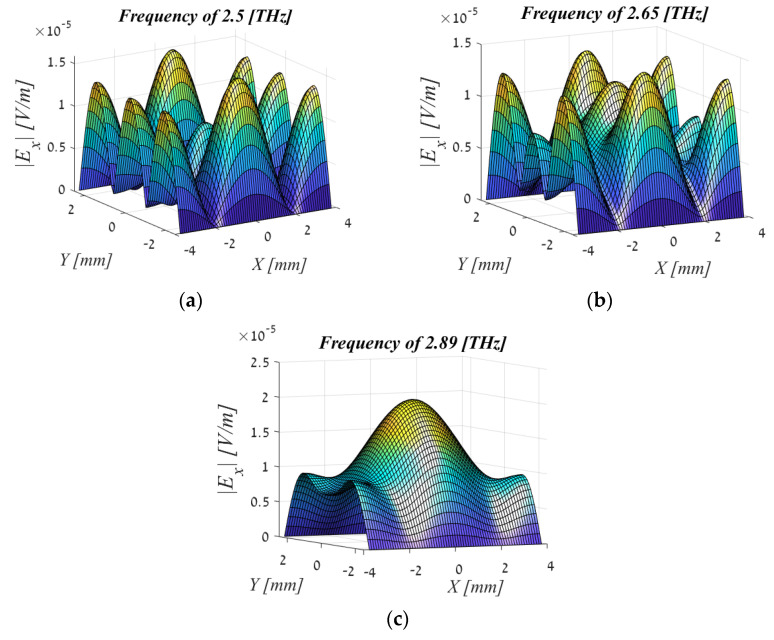
Spatial profile of the output radiation field: (**a**) f = 2.5 (THz), (**b**) f = 2.65 (THz), (**c**) f = 2.89 (THz). As can be seen at frequency 2.89 (THz), the energy is concentrated in the middle of the aperture and is maximum in size. Whereas, at a frequency of 2.65 (THz), the energy is smaller and what is important is that it is concentrated on the sides. At 2.5 (THz) the energy is even smaller. Since the concentration is on the sides and there is almost nothing in the middle of the aperture, we will perform the calculations for the frequency 2.89 (THz).

Analysis of the obtained radiation reveals that TE01, TE21, and TE23 waveguide modes are mainly excited by the electron beam; excitation of other waveguide modes is negligible. This means that the power is concentrated in those three modes. The radiation spectrum at the source output (exit of the waveguide) is built in MATLAB as a field profile based on a table of results from WB3D code at three important points that are demonstrated in Figure 3. Characteristic frequency points of about 2.89 THz (the peaks of TE01 and TE21 components), 2.5 THz (the peak of TE23 component), and 2.65 THz (the intermediate point where all the components considered have approximately equal spectral energy flux) can be marked at the radiation spectrum. Special attention is given to these characteristic frequency points in the following study of the transmission line. Spatial profiles of the output radiation field components at these characteristic frequency points are shown in Figure 4. Note that the output field keeps the boundary conditions at the walls of the rectangular waveguide in which it was excited. The shape of a field profile corresponds to the symmetry of the dominant waveguide component at the chosen characteristic frequency.

The magnitude of the energy and its distribution is easily distinguished from the profiles. At the center frequency, where even the maximum energy is obtained, a beautiful and symmetrical profile containing the majority of the energy is concentrated and transmitted in the middle of the aperture and fades at the edges. In addition, it can be seen that this profile is symmetrical to the quarter signal. We will discuss this in the next chapter when we discuss WDF results.

### 3.2. WDF Simulations

As a major part of the study, a special code was developed for the WDF. Currently, there is no universal standard code for calculating the WDF of an EM field, pulse, or beam. With an emphasis on two or three spatial dimensions, a universal code was developed that allows extension from 2 to the N dimension of WDF.

Due to the fact that our problem is based on a multiplicity of modes and multiplicity of frequencies, a code that would address all of the above and allow for expansion was required.

We chose a non-conventional way to carry out our frequency transformations. Many conventional practices only deal with one-dimensional WDF. These are described in the majority of articles. Additionally, they usually address each frequency individually; namely, each mode is calculated individually in order to get a general picture and it is necessary to superimpose everything. In this approach, the profile of the field is sampled. The field can have *N* dimensions at the desired resolution. The MI is achieved from that respective resolution. The suitability of each sample is tested with all its neighbors in each direction. As a result, a double-resolution is obtained. When controlling the resolution, the number of rays for each position of the field is upgradeable by changing resolution. At this point, the consideration of the resolution and direction of the future pattern is obtainable.

Therefore, regardless of the number of modes or frequencies that make up the field, everything is considered when the WDF amplitude is complex. At this point, we are able to monitor the phase accumulation (if necessary). Since the purpose of this work is to characterize the field in order to address or reinforce the further design of TL with desired mirrors, further details of the TL design are beyond the scope of this work.

In this work, it is important to plot the images in three dimensions rather than in the usual classical representation of Wigner because one gets a visualization and spatial distribution (mapping) of the THz signal. The field profile in K space gives an indication of the future propagation of the rays, which can then be tracked by RT.

**Figure 5:**
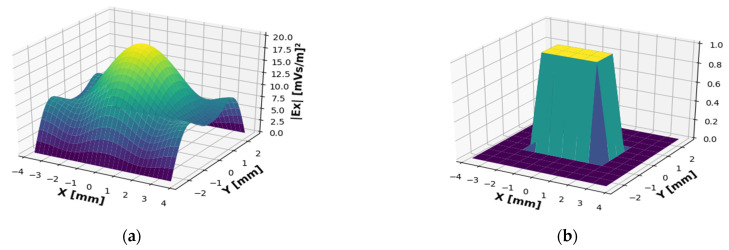
(**a**) Spatial profile of the output radiation field at the working frequency of 2.89 (THz) and the test signal and (**b**) the window function (test signal).

By using a universal 2D Python code that we have developed, a 5D WDF was obtained where the axes are *x*, *y*, *k_x_*, *k_y_*_,_ and the WDF is a complex function. The ***E_x_*** Field distribution at the output of a waveguide is demonstrated in Figure 5a together with the test signal (the window function) in Figure 5b.

**Figure 6:**
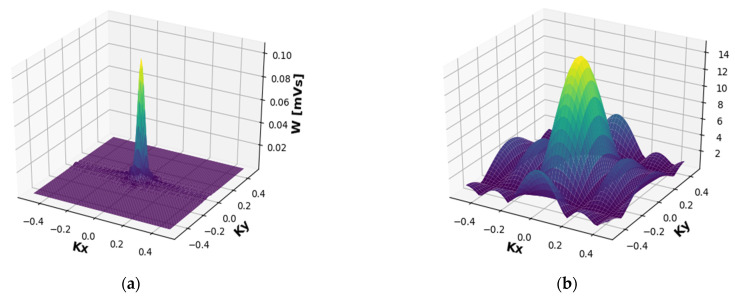
WDF from the center of the aperture—*W_x_* (*x* = 0, *y* = 0). (**a**) Of the radiation field at 2.89 (THz). (**b**) Of the window function. The energy is concentrated in the middle of the aperture.

Figure 6a shows the WDF of it in the middle of the aperture (coordinates *x* = 0 and *y* = 0). It can be shown that the energy is mostly concentrated in the center and therefore, by and large, propagates straight in the *z* direction. The spread of *k_x_* and *k_y_* angles is very small and therefore is negligible in the energy dispersion. This indicates that at this specific frequency the radiation goes forward along the *z*-axis. Consequently, this is the desired working frequency. At this frequency, most of the radiation is concentrated around the electron beam that is in the middle of the profile. In reality, a frequency of synchronization of the electron with an undulator is expressed.

The WDF repeats the ‘pattern’ of the original field, as can well be seen in Figure 6a. One may regard this as another proof of the necessity of this approach. Needless to say, the figures present the absolute value of WDF, while in practice each value is complex. The phase profile is not displayed because it is not needed in this work.

**Figure 7:**
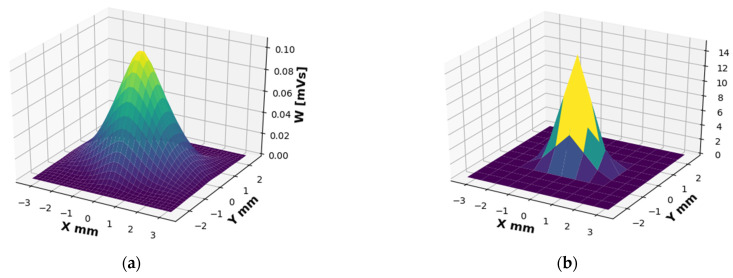
WDF from the entire aperture, only with rays propagating parallel to the *z*-axis, *W_x_* (*k_x_* = 0, *k_y_* = 0). (**a**) Of the radiation field at 2.89 [THz]. (**b**) Of the window function.

A profile of the field of rays parallel to the *z* axis ($k_{\left\{x,y\right\}}=0$) across the entire aperture is shown in Figure 7a. One can see the Gaussian behavior.

For a quick illustration, the code was activated on the test signal (window function). In the resulting simulation, we got a ‘perfect sync’ signal (Figure 6b) for the key coordinates (*x* = 0, *y* = 0). In addition, for all the rays that came out in parallel (*k_x_* = 0, *k_y_* = 0) we got a ‘perfect triangle’ signal (Figure 7b), which perfectly matches the theory and analytical calculation. At this time, the code is not published and will be used for future research.

For convenience, the field and window function images are facing each other. It is easy to see the correspondence between the field and the window function and their WDF components. This emphasizes the correctness of our code.

## 4. Discussion

A visualization of the EM field at the central frequency of 2.89 THz at the entrance to TL was obtained including both a spectral representation and a representation in terms of rays. The results at other frequencies are less important and therefore are not included in the publication.

It is important to analyze plot images in three dimensions rather than in the usual classical 2D Wigner representation. This provides an indication of the resultant rays and allows one to see the profile and prediction of the rays in the RT.

This article focused on the ***E_x_*** field at a central frequency of 2.89 THz. In this frequency the energy is maximal and the field consists of three main modes, as was mentioned above. The EM field representation mentioned may be easily expanded to more mode representations as well as an addition to the TM modes to complete the ***E_x_*** field. Note that the contribution of TM-Modes is negligible. One may also expand the research by adding an *E_y_* field (containing all its components) and examining all changes due to its contribution. To do this, all the steps done for the *E_x_* field must be repeated. Pre-preparations have shown that the contribution of the *E_y_* field is relatively small. Indeed, it is also possible to extend to the time component of the WDF, which is a basis for a study in future research.

## Data Availability

The data presented in this study are available on request from the corresponding author. The data are not publicly available due to belonging to the research and development of part of a compact particle accelerator at the Schlesinger Family Center for Compact Accelerators, Radiation Sources and Applications (FEL).
